# Identification of Ferroptosis-Associated Genes in Prostate Cancer by Bioinformatics Analysis

**DOI:** 10.3389/fgene.2022.852565

**Published:** 2022-07-04

**Authors:** Qijun Wo, Zhenghong Liu, Linyi Hu

**Affiliations:** ^1^ Urology and Nephrology Center, Department of Urology, Zhejiang Provincial People's Hospital (Affiliated People's Hospital, Hangzhou Medical College), Hangzhou, China; ^2^ The Second Clinical Medical College, Zhejiang Chinese Medical University, Hangzhou, China

**Keywords:** ferroptosis potential index, prostate cancer, tumor mutation burden, BCR-free survival, drug resistance

## Abstract

**Background:** In order to reveal the functions of ferroptosis in prostate cancer (PCa), a ferroptosis potential index (FPI) was built. This study researched the influence of ferroptosis on gene mutations, various cellular signaling pathways, biochemical recurrence (BCR), and drug resistance in both FPI-high and FPI-low groups.

**Methods:** RNA-seq, somatic mutation data, and clinical data were obtained from The Cancer Genome Atlas (TCGA). FPI values were calculated. All samples were divided into FPI-high and FPI-low groups. The BCR-free survival rate, tumor mutation burden (TMB) value, cellular signaling pathway, differentially expressed genes (DEGs), and drug resistance in the two FPI groups were identified. Human PCa cells, LNCaP, were treated with ferroptosis inducer erastin or inhibitor ferrostatin-1. The expression of hub genes was detected by qRT-PCR and Western blot.

**Results:** A high FPI level was significantly related to poor BCR-free survival. Also, higher TMB value was found in the FPI-high group, and FPI was shown to be associated with gene mutations. Then, genes in both groups were revealed to be enriched in different pathways. A total of 310 DEGs were identified to be involved in muscle system processes and neuroactive ligand–receptor interactions. A total of 101 genes were found to be related to BCR-free survival, and a protein–protein interaction (PPI) network was constructed. Two sub-modules were identified by MCODE, and eight hub genes were screened out, among which *SYT4* had higher expression levels and poorer BCR-free survival in the FPI-high group, while the remaining hub genes had lower expression levels and poorer BCR-free survival. Drug sensitivity was revealed to be different in the two groups by study on the IC_50_ data of different molecules and ferroptosis regulator gene (FRG) expressions. Finally, erastin increased the expression of SYT4 in LNCaP and decreased the expression of the other four genes (ACTC1, ACTA1, ACTN2, and MYH6), while ferrostatin-1 led to the opposite results. The molecular experimental results were consistent with those of bioinformatics analysis, except TNNI1, TNNC2, and NRAP.

**Conclusion:** The current research depicted the ferroptosis level and FRGs in PCa. Ferroptosis was related to TMB value, BCR-free survival, and drug resistance. This study will be beneficial to further research studies on ferroptosis-related molecular mechanisms.

## Introduction

Prostate cancer (PCa) is one of the world’s most common male malignancies (Luo et al., 2020). The occurrence of PCa is not only related to genetic factors but also closely associated with environmental factors (pathogenic microorganism infection, diet, bad habits, etc.). The number of diagnosed cases of PCa reached 190,000 and the death toll rose to 30,000 by 2020 because of extensive use of PSA screening (Siegel et al., 20202020). The risk stratification of PCa determines the treatment modalities in clinical practice (Herden et al., 2017). Nowadays, treatment methods for PCa include surgery, radiotherapy, chemotherapy, and so on (Tosoian et al., 2017). Unsatisfactorily, biochemical recurrence (BCR) still occurs after surgery, and detectable tumor recurrence was found in about 30% of such patients (Ward et al., 2003; Stephenson et al., 2006; Zanaty et al., 2018). Therefore, it is of great importance to find a new treatment for PCa.

In 2012, Dixon found that erastin can induce a cell death pattern in tumor cells characterized by mitochondrial shrinkage and increased double-membrane density, iron-dependent lipid peroxidation, and regulation by the cystine transport pathway, naming it ferroptosis (Dixon et al., 2012). Ferroptosis differs from apoptosis, necrosis, and autophagy in morphology, biochemistry, and gene regulation. It requires no energy consumption, is not inhibited by apoptosis inhibitors, and has no calcium overload in the cell. Furthermore, ferroptosis plays a pivotal role in inhibiting tumorigenesis by removing the damaged cells (Fearnhead et al., 2017) and has come to be a vital approach for the treatment of a number of malignant tumors, such as kidney cancer, pancreatic cancer, breast cancer, and PCa, which are induced by small-molecule drugs (Yang et al., 2014; Eling et al., 2015; Louandre et al., 2015; Yu et al., 2019; Ghoochani et al., 2021). Moreover, ferroptosis regulator genes (FRGs) and ferroptosis have been certified to be related to drug resistance (Lu et al., 2017). The ferroptosis potential index (FPI) was built to evaluate ferroptosis levels in this study. The exploration of ferroptosis is expected to inspire new treatment strategies for tumors.

We performed an overall analysis of FRGs in PCa, calculated the FPI with the expression of FRGs, and analyzed the relationship between gene mutations and ferroptosis. As a result, we discovered that ferroptosis was related to various cellular signaling pathways, BCR, and drug resistance. The current study highlights the key roles of ferroptosis in PCa, which will contribute to further research studies on the molecular mechanisms and theoretical progress of ferroptosis, thereby providing a new idea for the treatment of cancer.

## Methods

### Data Download

The Cancer Genome Atlas (TCGA) is a cancer genome database that facilitates cutting-edge cancer studies. There were RNA-seq, somatic mutation, and clinical data of PCa patients (TCGA-PRAD) from the TCGA database (https://portal.gdc.cancer.gov/). These samples included 489 cancer tissues and 51 adjacent tissues. The RNA-seq counts were normalized by the edgeR package (version 3.32.1) (Robinson et al., 2010; McCarthy et al., 2012) and were used as the gene expression data. GSE46602 from the Gene Expression Omnibus (GEO) database was downloaded and used to verify the relationship between FPI level and BCR-free survival. There were 21 patients with disease recurrence and 14 without recurrence. The flowchart of TCGA-PRAD data analysis is provided as Supplementary Figure S1.

### The Establishment of FPI Model

The FPI model representing the ferroptosis level was established with the expression data of FRGs, including the positive components (PCs) of lysophosphatidylcholine acyltransferase 3 (*LPCAT3*), nuclear receptor coactivator 4 (*NCOA4*), acyl-CoA synthetase long-chain family member 4 (*ACSL4*), arachidonate 15-lipoxygenase (*ALOX15*), solute carrier family 7 member 11 (*SLC7A11*), glutathione peroxidase 4 (*GPX4*), solute carrier family 3 member 2 (*SLC3A2*), NADPH oxidase 1 (*NOX1*), erythroid 2 like 2 (*NFE2L2*), NADPH oxidase 4 (*NOX4*), and NADPH oxidase 5 (*NOX5*) and the negative components (NCs) of farnesyl-diphosphate farnesyltransferase 1 (*FDFT1*), coenzyme Q10 production A (*COQ10A*), 3-hydroxy-3-methylglutaryl coenzyme A reductase (*HMGCR*), and coenzyme Q10 production B (*COQ10B*) identified in existing research studies (Liu et al., 2020). The enrichment score (ES) was calculated using the R package “GSVA” (ssGSEA) in the gene set. The FPI was obtained according to the formula FPI = ES_PC_-ES_NC_ (Liu et al., 2020). Based on the median FPI (−0.57), we divided samples into the FPI-high group and the FPI-low group.

### Tumor Mutation Burden Value Estimation

TMB value refers to the amount of mutations per megabyte, including the amount of base substitution insertions or deletions and gene coding errors (Luo et al., 2020). TMB was obtained by dividing the frequency of mutations according to the size of the encoding region of the target gene. 38 MB is regularly used based on the length of the human exon. TMB = the total mutation number/38.

### Relationship Between TMB Value and FPI

The somatic mutation data from the TCGA database were analyzed and then visualized using the “maftools” in R package to obtain the top 20 mutation genes between the FPI-high and FPI-low groups (Luo et al., 2020). Analysis of the TMB value between the two groups was performed using the R package.

### Gene Set Enrichment Analysis

GSEA was conducted between the FPI-high group and the FPI-low group using GSEA v4.1.0 software in order to confirm the pathways related to ferroptosis in PCa.

### Identification of Differentially Expressed Genes

FPI-related DEGs were identified using DESeq2, and all DEGs with |logFC| > 1 and padj <0.05 were exported. Finally, the “pheatmap” R package was used to show the volcano plot of DEGs.

### Functional Enrichment Analysis of FRGs

“clusterProfiler” in the R package was used in the study to conduct the gene ontology (GO) pathway enrichment analysis and Kyoto Encyclopedia of Genes and Genomes (KEGG) pathway analysis of FRGs (Yu et al., 2012).

### Prognostic Evaluation of Ferroptosis-Related DEGs and Protein–Protein Interaction Network Construction

FPI-related DEGs were analyzed using univariate Cox regression analysis. Genes significantly associated with BCR were identified with a *p* value <0.05. Then, the PPI network was built using the STRING database. The number of core gene nodes was visualized using Cytoscape software in the R package (Shannon et al., 2003; Szklarczyk et al., 2015). MCODE was used to analyze the sub-modules of the PPI network, with an MCODE score >4 and the number of nodes >4. There were eight hub genes selected by the CytoHubba in the Cytoscape software.

### Relationship Between FRGs and Drug Sensitivity

In order to identify the relationship between ferroptosis-related genes and small-molecule drugs, a web tool GSCALite (http://bioinfo.life.hust.edu.Cn/web/GSCALite/) was applied to analyze the relationship between the expression of FPI-related DEGs and different drug IC_50_ in PCa cell lines (Sun et al., 2020).

### Cell Culture and Grouping

Human PCa cells, LNCaP, were purchased from the Chinese Academy of Sciences cell bank. LNCaP cells were cultured in RPMI-1640 medium (Gibco) containing 10% fetal bovine serum, in which the concentration of penicillin and streptomycin (P/S) was 1%. LNCaP cells were cultured under conventional conditions. LNCaP cells were treated with ferroptosis inducer erastin (10µM, 6 h) or ferroptosis inhibitor ferrostatin-1 (2 µM, 6 h). Grouping: Control, LNCaP + erastin, LNCaP + ferrostatin-1. In order to examine the change in ferroptosis level, the ROS level of LNCaP was measured by 2′, 7′-dichlorodihydrofluorescein diacetate (H (2) DCFDA).

### qRT-PCR

According to the instructions, the RNA of LNCaP cells was extracted with the TRIzol Kit (Invitrogen), and then the cDNA was obtained with the ReverTra Ace qPCR RT Kit (Toyobo). Then, the relative expression level of mRNA was detected on an ABI 7900 real-time fluorescence quantitative PCR instrument (ABI) using the real-time fluorescence qPCR detection kit (Roche). β-actin was an internal reference gene. qRCR-related primers are listed in Table 1.

**TABLE 1:** qRCR related primers.

Gene	Forward primer	Reverse primer
ACTC1	CTA​AGG​GGC​TGG​GTT​TCT​TG	ACA​GAG​AAG​GGC​AGA​GGA​AT
ACTA1	CTT​TGG​GCT​CCT​TTA​CCT​GG	CTA​GCG​CTA​GTT​TGG​AAG​GG
ACTN2	TTA​GAG​AGA​CCA​GGC​ACA​CA	GTG​ACC​TCT​TGA​TGC​GGA​AT
TNNI1	GAT​GGA​TGC​TTT​CAC​GTT​GC	GGG​GCA​TTC​TTA​TTG​GGA​GG
TNNC2	GGG​GCA​GTA​ACT​TGG​ATG​TT	GCC​CTC​CCC​TTT​CAA​GAA​AA
SYT4	AAA​GCA​AAT​CCA​GCC​TGT​GA	TGG​CTG​TGT​TTT​CCA​TCG​AA
MYH6	GGT​GCA​GCA​AAA​CAG​GAT​TC	CAT​GTC​CAG​CAC​TCA​GAG​TC
NRAP	ATT​CGT​GGT​GAG​GCT​GTT​TT	TAA​GCA​CAG​CGT​CAG​GAT​TC

### Western Blot

Western blot was performed according to the steps described in the literature to detect the protein expression level of LNCaP cells (Sur et al., 2019). GAPDH was an internal reference. The antibodies used were purchased from Thermo Fisher Scientific (CN): ACTC1 (#PA5-21396), ACTA1 (#MA5-37536), ACTN2 (#PA5-27863), TNNI1 (#701585), TNNC2 (#PA5-76253), SYT4 (#MA5-26214), MYH6 (#MA5-24631), and NRAP (#MA5-24402).

### Statistical Analysis

This study used the “survival” package to conduct Kaplan–Meier analysis in order to describe the BCR-free survival curve, and then the significance was identified by the log-rank test. The R codes for bioinformatics analysis is provided in Supplementary Material. The differences between the two groups were analyzed using Student’s *t* test. *p*-value *<* 0.05 was judged to be statistically significant.

## Results

### Ferroptosis Level of PCa

**FIGURE 1:**
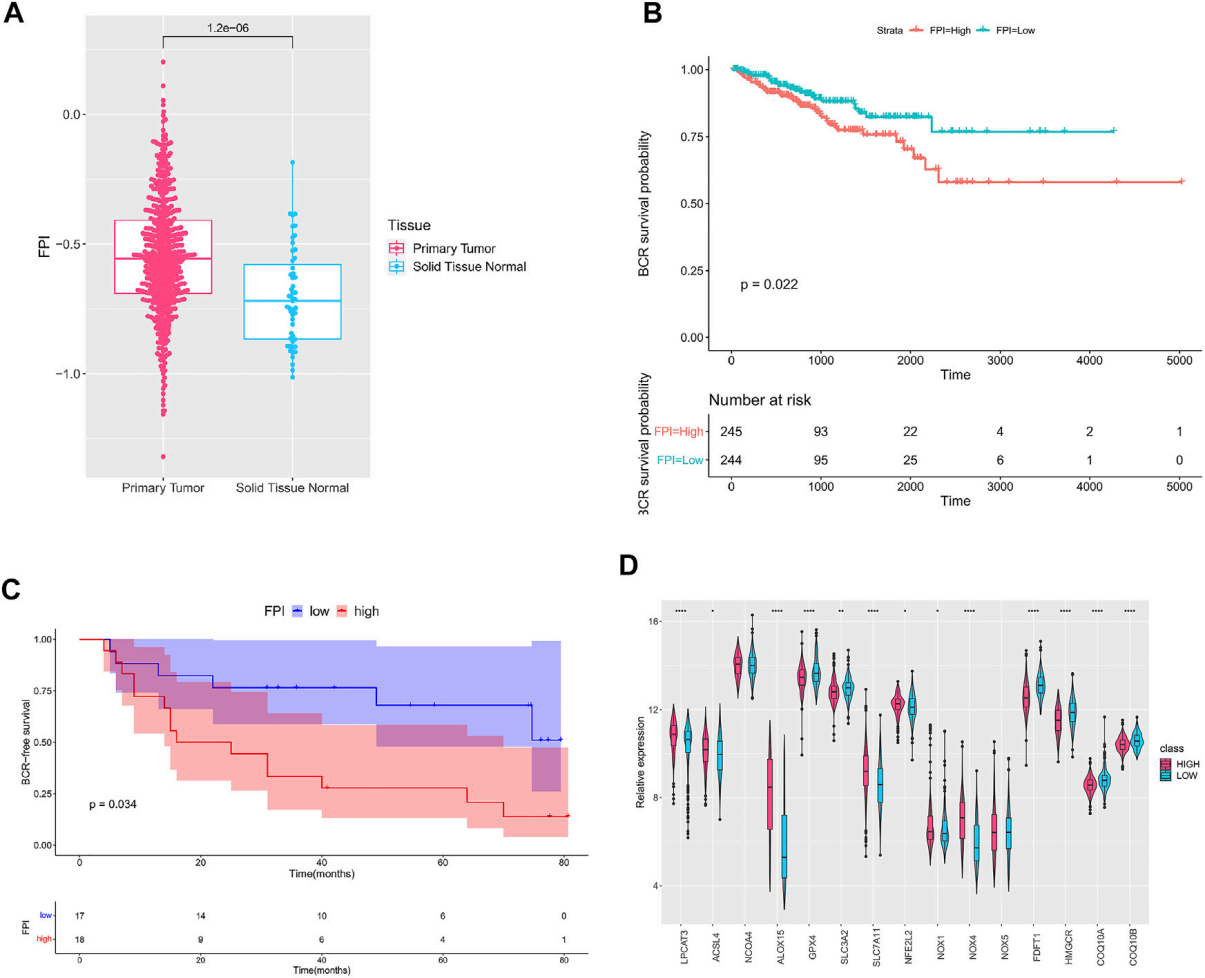
Ferroptosis in PCa. **(A)** FPI in tumor and adjacent tissues; **(B)** Kaplan–Meier curve of the FPI-high group and FPI-low group in TCGA; **(C)** Kaplan–Meier curve of the FPI-high group and FPI-low group in GSE46602; **(D)** relative expression of FRGs in two groups.

A total of 15 genes were identified as FRGs by previous studies, and they were shown to play a key role in the regulation of ferroptosis, including PCs: *NCOA4*, *LPCAT3*, *GPX4*, *ACSL4*, *SLC3A2*, *ALOX15*, *SLC7A11*, *NOX1*, *NFE2L2*, *NOX4*, and *NOX5* and NCs: *FDFT1*, *COQ10A*, *COQ10B*, and *HMGCR* (Liu et al., 2020). To further understand the functions of ferroptosis in PCa, the FPI was calculated by ssGSEA based on the ES of PCs minus that of NCs. The results obtained in the current study indicated that the FPI of tumor samples was obviously higher than that of adjacent tissues (Figure 1A). Based on the FPI median value, all tumor samples were segmented into two different groups, with 245 included in the FPI-high group and 244 in the FPI-low group. Clinical information in the FPI-low group and FPI-high group is displayed in Table 2. Poorer BCR-free survival was revealed in the FPI-high group than in the FPI-low group according to results of the Kaplan–Meier curve, with *p* value = 0.022 (Figure 1B). In other words, higher FPI predicted poorer prognosis. Meanwhile, GSE46602 was used to test the relationship between FPI level and BCR-free survival. It was also found that high FPI predicted poor prognosis (Figure 1C). On the other hand, gene expression analysis of the 15 FRGs revealed that there were significant differences in 13 of 15 gene expression between the FPI-high and FPI-low groups (Figure 1D).

**TABLE 2:** Clinical information in the FPI-low group and FPI-high group.

	FPI-low (*N* = 244)	FPI-high (*N* = 245)
Age		
≤ 60	127	94
>60	117	151
Gleason score		
≤ 6	33	13
7	135	111
8–10	76	121
pathologic_T		
T2	120	68
T3	115	168
T4	4	7
Missing	5	2
pathologic_N		
N0	168	171
N1	31	47
Missing	45	27

### Association of FPI and TMB

**FIGURE 2:**
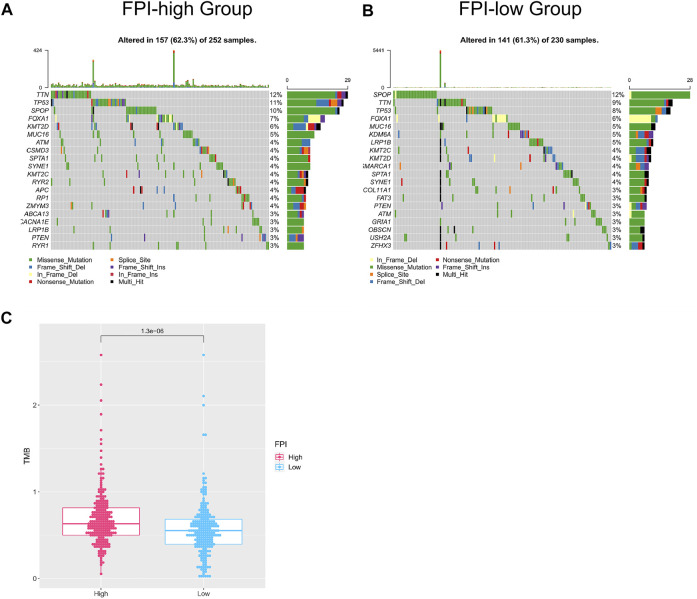
Mutated genes in the FPI-high group and FPI-low group. **(A,B)** Waterfall plot of the top 20 mutated genes (*X*-axis for samples, *Y*-axis for mutated genes, different colors represent mutation types); **(C)** TMB of the FPI-high and FPI-low groups.

We analyzed the correlation between FPI and somatic gene mutations. First of all, the whole-exome sequencing data of samples (FPI-high group and FPI-low group) were analyzed to provide a comprehensive mutation presentation. The waterfall plot showed mutational categories for each sample in the FPI-high group and the top 20 mutated genes (Figure 2A), namely, *TTN*, *TP53*, *SPOP*, *FOXA1*, *KMT2D*, *MUC16*, *ATM*, *CSMD3*, *SPTA1*, *SYNE1*, *KMT2C*, *RYR2*, *APC*, *RP1*, *ZMYM3*, *ABCA13*, *CACNA1E*, *LRP1B*, *PTEN*, and *RYR1*. Figure 2B showed the results of the FPI-low group, with the top 20 mutated genes, namely, *SPOP*, *TTN*, *TP53*, *FOXA*, *MUC16*, *KDM6A*, *LRP1B*, *KMT2C*, *KMT2D*, *SMARCA1*, *SPTA1*, *SYNE1*, *COL11A1*, *FAT3*, *PTEN*, *ATM*, *GRIA1*, *OBSCN*, *USH2A*, and *ZFHX3*. The TMB was calculated with the gene mutation frequency divided by 38 MB, which is also defined as the mutation frequency (Lv et al., 2020). To investigate the relationship between FPI and TMB, TMB values of FPI-high and FPI-low groups were calculated. The TMB value in the FPI-high group (a mean of 0.68) was revealed to be obviously higher than that in the FPI-low group (a mean of 0.58) (Figure 2C). That means the gene mutation frequency of the FPI-high group was higher than that of the FPI-low group.

### Relationship Between Cellular Signaling Pathways and FPI

**FIGURE 3:**
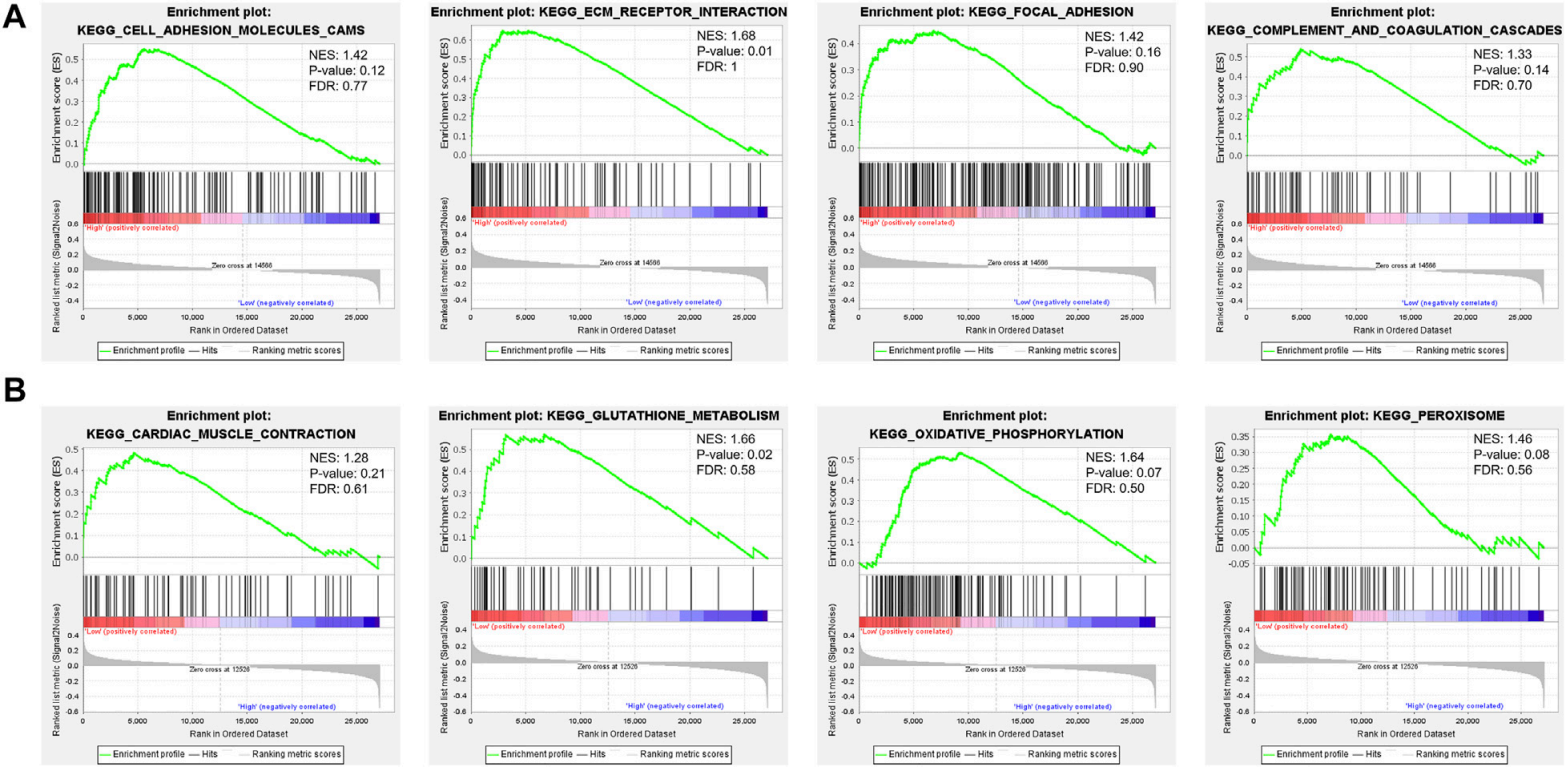
GSEA of FPI-high and FPI-low groups. **(A)** FPI-high group; **(B)** FPI-low group.

To carry out a profound study of the relationship between cellular signaling pathways and the FPI, we performed GSEA based on the transcriptome of FPI-high and FPI-low groups. It was found that some pathways in KEGG, such as cell adhesion molecules cams, focal adhesion, complement, ECM receptor interaction, and coagulation cascades, were usually enriched in the FPI-high group (Figure 3A), whereas cardiac muscle contraction, glutathione metabolism, oxidative phosphorylation, and peroxisome pathways were enriched in the FPI-low group (Figure 3B).

### Recognition of DEGs Between FPI-High Group and FPI-Low Group

**FIGURE 4:**
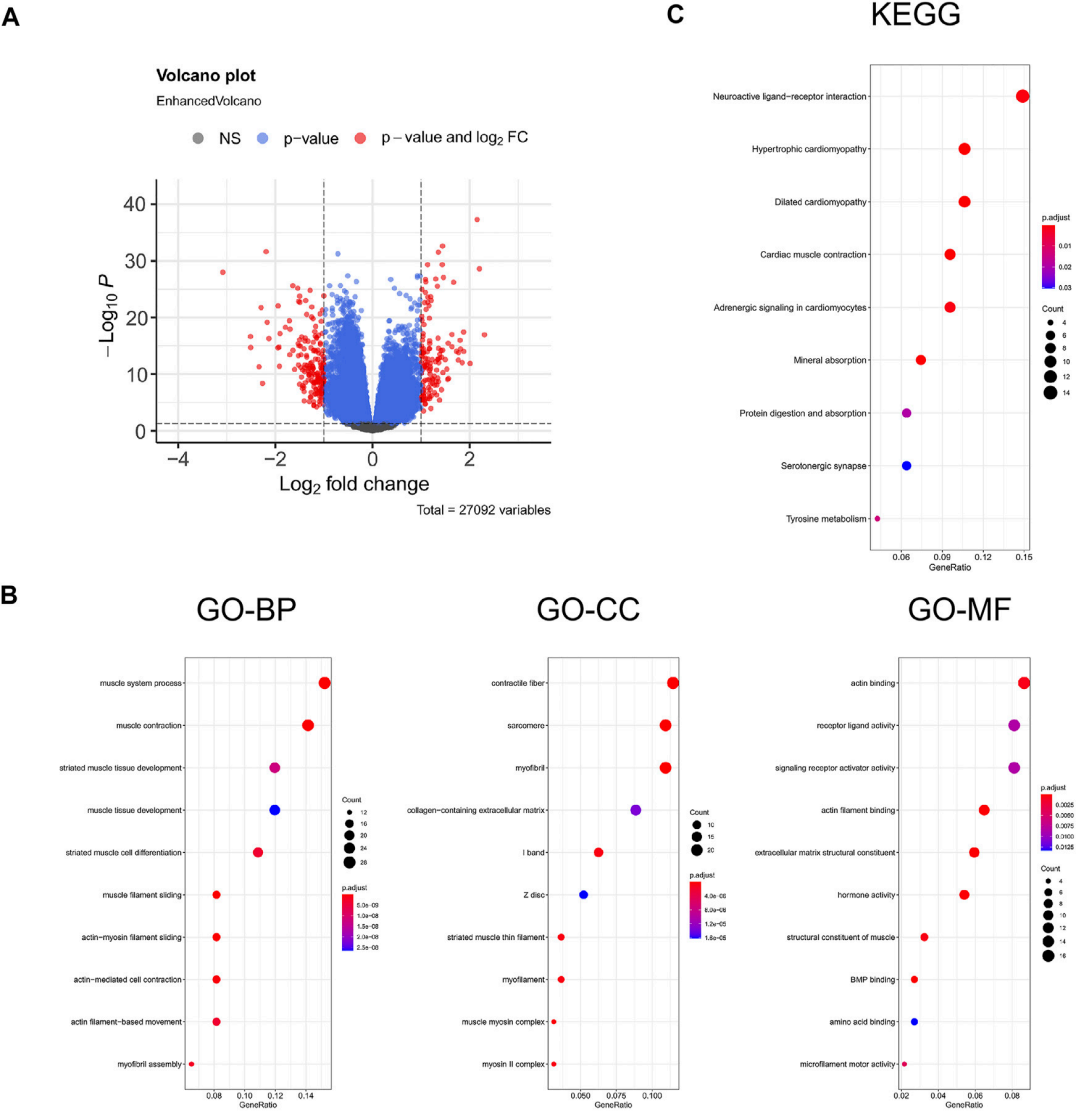
Enrichment analysis for the DEGs. **(A)** Volcano plot of DEGs, red represents the fold change and *p*-value meets the conditions, and blue represents that only *p*-value meets a condition; **(B)** GO functional analysis; **(C)** KEGG pathway. GeneRatio represents the ratio of enriched genes to inputted genes, BP represents biological processes, CC represents cellular component, and MF represents molecular function.

FPI-associated DEGs in PCa were analyzed using DESeq2 with |logFC| >1 and padj <0.05. 310 DEGs were identified in all, including 135 upregulated genes and 175 downregulated genes. The volcano plot is displayed in Figure 4A. In order to confirm the biological functions and roles of DEGs, clusterProfiler was used in the current study for conducting GO and KEGG analyses. GO analysis indicated that these DEGs were involved in muscle system processes, muscle contraction, striated muscle tissue development, and so on (Figure 4B). KEGG analysis revealed that these DEGs were related to neuroactive ligand–receptor interaction and hypertrophic cardiomyopathy (Figure 4C).

### Prognosis Analysis of FPI-Associated DEGs

**FIGURE 5:**
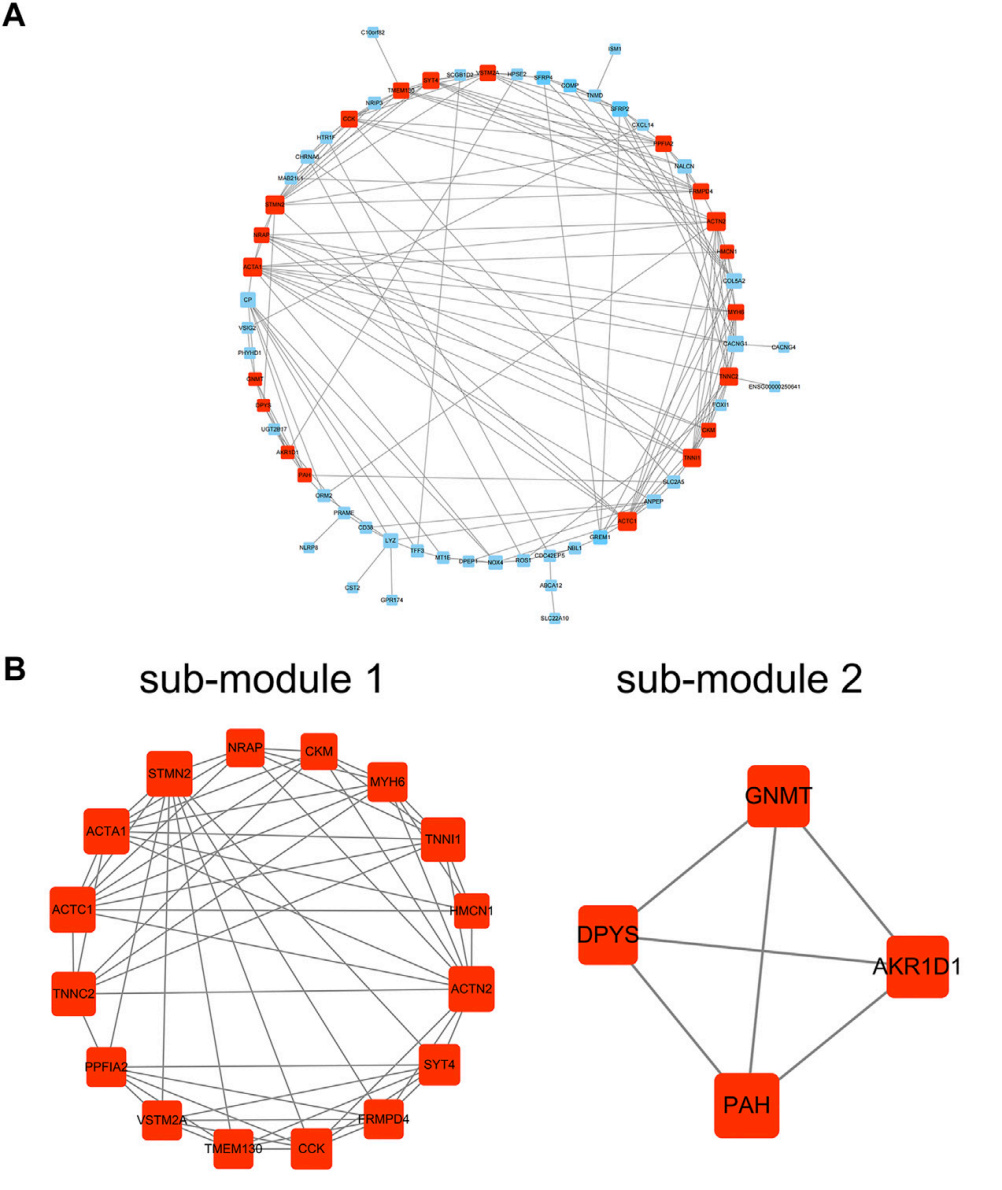
PPI network analysis. **(A)** PPI network; **(B)** sub-module 1 and sub-module 2. PPI: protein–protein interaction.

**FIGURE 6:**
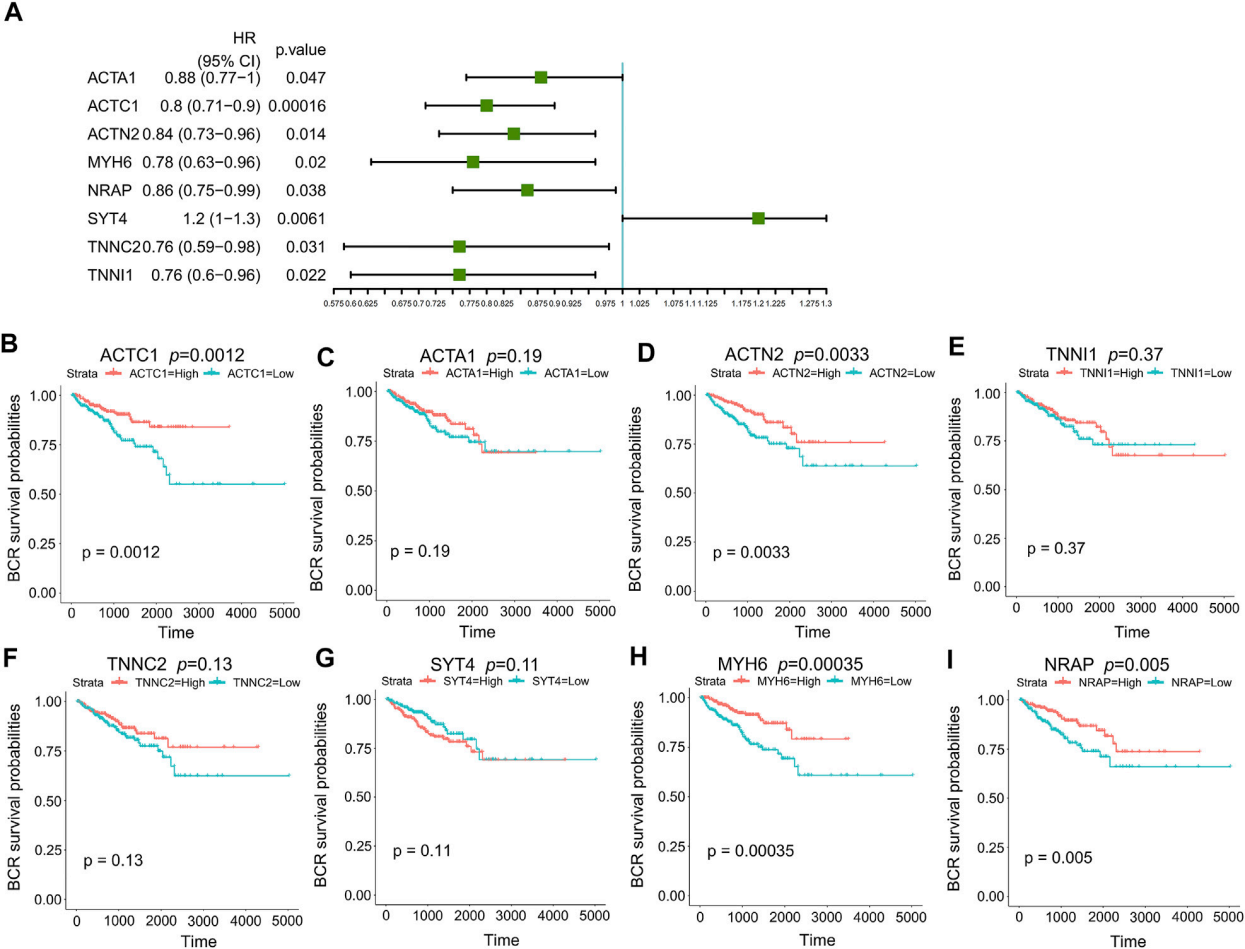
Prognosis analysis of eight hub genes. **(A)** *p* value and HR of eight hub genes in univariate Cox regression analysis; **(B)** Kaplan–Meier curves of *ACTC1*; **(C)** Kaplan–Meier curves of *ACTA1*; **(D)** Kaplan–Meier curves of *ACTN2*; **(E)** Kaplan–Meier curves of *TNNI1*; **(F)** Kaplan–Meier curves of *TNNC2*; **(G)** Kaplan–Meier curves of *SYT4*; **(H)** Kaplan–Meier curves of *MYH6*; **(I)** Kaplan–Meier curves of *NRAP*. HR: hazard ratio.

101 candidates related to BCR-free survival were screened out based on univariate Cox regression analysis, with a *p* value < 0.05. In addition, we built a PPI network to find the hub genes. The network diagram is displayed in Figure 5A which was optimized using Cytoscape software. The sub-module analysis of the PPI network was performed through MCODE. As shown in Figure 5B, there are two sub-modules, sub-module 1 (16 nodes and 60 edges, score = 8) and sub-module 2 (four nodes and six edges, score = 4). Then, GO and KEGG analyses were performed on the two sub-modules. The modules were associated with actin-myosin filament sliding, muscle filament sliding, actin filament–based movement, actin-mediated cell contraction, etc. (Table 3). Hub genes were identified using cytoHubba Cytoscape software (maximally connected components (MCC) and EPC method). The top 10 genes in the PPI network are shown in Table 4 which were ranked by the MCC method. The result of the EPC method is shown in Table 5. There are eight hub genes, namely, *ACTA1*, *ACTC1*, *ACTN2*, *MYH6*, *NRAP*, *SYT4*, *TNNC2*, and *TNNI1*. Figure 6A shows the univariate Cox regression analysis results of eight hub genes, and Kaplan–Meier analysis was used in this study to evaluate the prognostic effect of the eight hub genes (Figures 6B–I). We found that the high-expressed gene (*SYT4*) and low-expressed genes (other seven genes) in the FPI-high group had poor BCR-free survival. The details of the eight hub genes are listed in Table 6.

**TABLE 3:** GO and KEGG pathway enrichment analysis of DEGs in two modules.

Pathway description	*p*.adjust	Genes
Muscle filament sliding	3.74E-10	MYH6, TNNI1, ACTN2, TNNC2, ACTC1, and ACTA1
Actin-myosin filament sliding	3.74E-10	MYH6, TNNI1, ACTN2, TNNC2, ACTC1, and ACTA1
Actin-mediated cell contraction	2.80E-07	MYH6, TNNI1, ACTN2, TNNC2, ACTC1, and ACTA1
Actin filament-based movement	5.76E-07	MYH6, TNNI1, ACTN2, TNNC2, ACTC1, and ACTA1
Myofibril assembly	8.98E-07	MYH6, ACTN2, NRAP, ACTC1, and ACTA1
Cardiac muscle contraction	0.045027	MYH6 and ACTC1
Hypertrophic cardiomyopathy	0.045027	MYH6 and ACTC1
Dilated cardiomyopathy	0.045027	MYH6 and ACTC1

**TABLE 4:** Top 10 genes ranked by the MCC method.

Rank	Name	Score
1	ACTA1	1812
2	TNNI1	1808
3	ACTN2	1807
4	MYH6	1800
5	ACTC1	1,580
6	CKM	1,440
7	TNNC2	848
8	NRAP	846
9	SYT4	757
10	FRMPD4	756

**TABLE 5:** Top 10 genes ranked by the EPC method.

Rank	Name	Score
1	ACTN2	24.182
2	ACTC1	23.83
3	ACTA1	23.515
4	TNNI1	23.472
5	STMN2	23.198
6	TNNC2	22.705
7	NRAP	22.283
8	MYH6	22.166
9	PPFIA2	22.066
10	SYT4	21.62

**TABLE 6:** Information of eight hub genes.

Gene	FPI-low group	FPI-high group	log2FC	*p* value
ACTC1	9.212118219	8.406873871	−1.04801	6.15E-09
ACTA1	7.235322213	6.497485355	−2.29293	4.81E-25
ACTN2	6.493852102	5.820841751	−1.13328	2.92E-08
TNNI1	8.25008438	7.789582888	−1.49994	2.25E-27
TNNC2	5.980030576	5.506088113	−1.04774	1.14E-12
SYT4	5.519441595	6.111665312	1.875549	2.78E-20
MYH6	4.970200339	4.299258419	−1.41282	2.01E-09
NRAP	5.550597763	4.909468861	−1.91628	1.64E-13

FC: fold change.

### Ferroptosis and Drug Sensitivity

**FIGURE 7:**
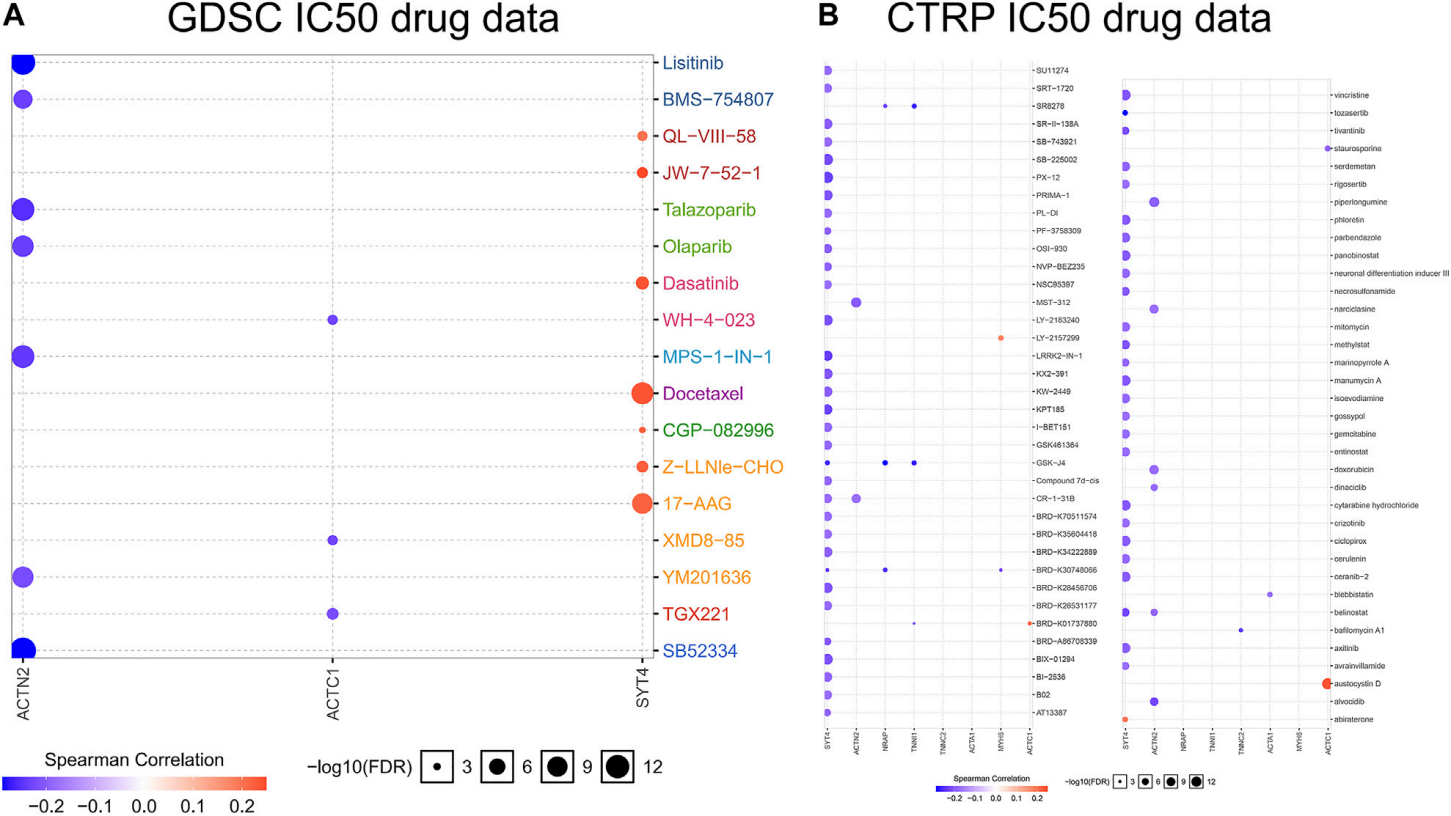
Bubble heatmap which was obtained to describe the correlation between IC_50_ data of different molecule drugs and the eight hub gene expression profile in PCa cell lines. *X*-axis for FRGs, *Y*-axis for different drugs. **(A)**: GDSC IC50 drug data; **(B)**: CTRP IC50 drug data.

To deeply recognize the relationship between drug sensitivity and ferroptosis, the GSCALite online tool was used to identify the relationship between IC_50_ data of different molecules and eight hub gene expressions in the PCa cell lines, indicating that ferroptosis regulation could influence the therapeutic effectiveness of tumor treatments. There are two databases, GDSC and CTRP. The positive correlation implies that highly expressed genes are resistant to the drugs, and vice-versa. Among the eight hub genes, drug sensitivity was associated with *SYT4* expression in the highest number of drugs, whereas drug sensitivity was associated with *TNNC2* and *ACTA1* expression in the lowest number (Figure 7). *SYT4* was negatively correlated with the drug sensitivity of most molecules, indicating that *SYT4* enables ferroptosis to play a promoting role in drug treatment. In other words, these results indicate that FRGs are related to the sensitivity of multiple drugs.

### Gene Expression Validation

**FIGURE 8:**
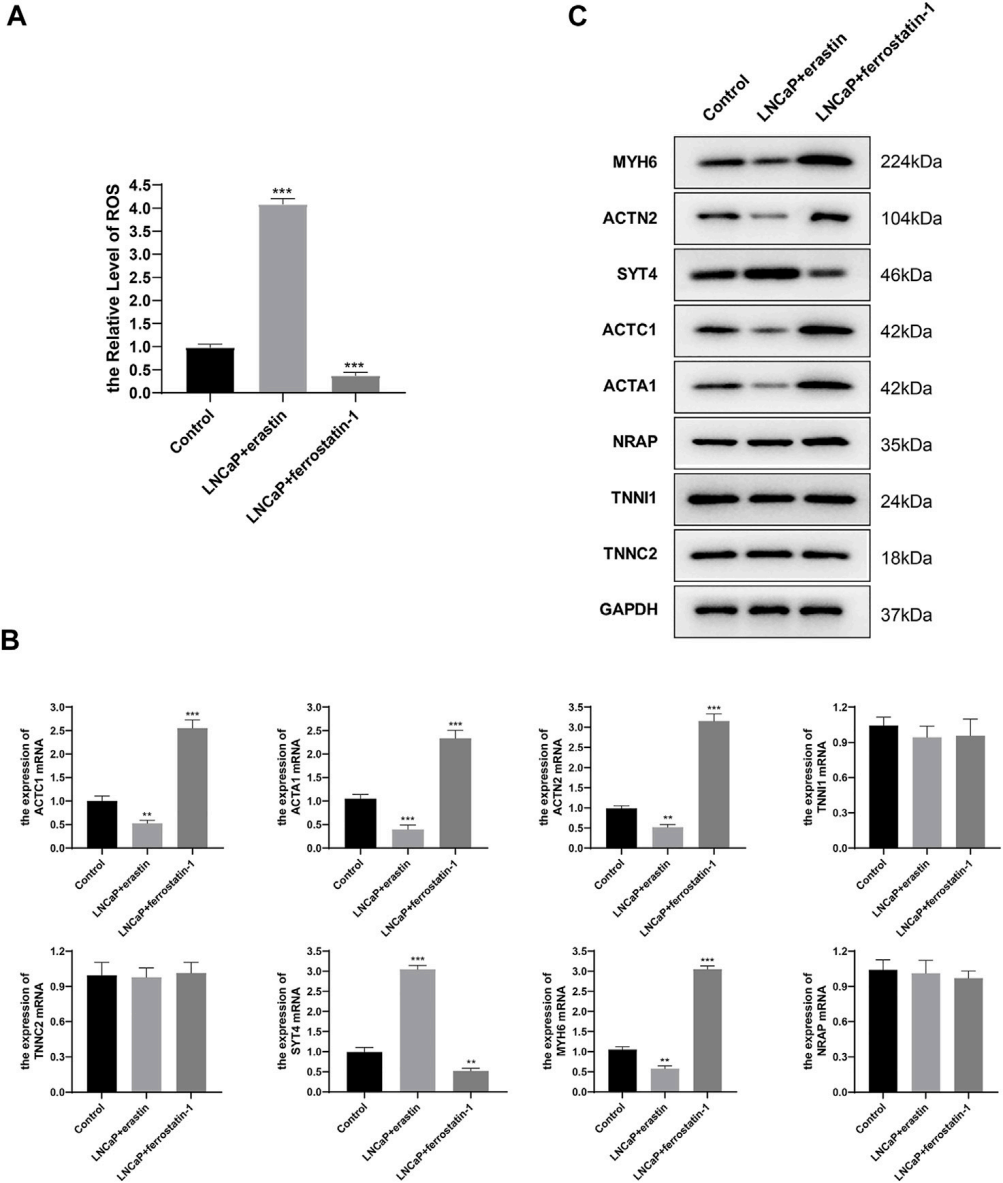
Hub gene expression in LNCaP. **(A)** H (2)DCFDA probe was used to determine the ROS level of LNCaP; **(B)** mRNA relative expression levels were determined by qRT-PCR; **(C)** Protein expression was determined by Western blot.

It is known that ferroptosis inducers can significantly increase FPI, while ferroptosis inhibitors can significantly reduce FPI (Liu et al., 2020). The H(2) DCFDA probe was used to determine the ROS level. It was found that ferroptosis inducer erastin induced ROS accumulation and increased ferroptosis level; ferroptosis inhibitor ferrostatin-1 decreased the level of ROS and attenuated ferroptosis level (Figure 8A). qRT-PCR results showed that the relative expression levels of SYT4 mRNA increased, and the relative expression levels of ACTC1, ACTA1, ACTN2, and MYH6 mRNA decreased after erastin treatment of LNCaP cells; after treatment of LNCaP cells with ferrostatin-1, the relative expression levels of SYT4 mRNA decreased, and the relative expression levels of the other four genes increased; the relative expression levels of TNNI1, TNNC2, and NRAP had no significant change in the three groups (Figure 8B). The protein expression of eight genes was examined by Western blot. The results of protein expression were consistent with those of qRT-PCR (Figure 8C). The abovementioned results were consistent with bioinformatics analysis results, except for TNNI1, TNNC2, and NRAP.

## Discussion

Ferroptosis is driven by the destruction of cell membranes caused by the inactivation of GPX4 (Feng and Stockwell, 2018). Past studies indicate that ferroptosis is closely correlated with tumorigenesis and plays a crucial role in tumor treatment (Yang et al., 2014; Shen et al., 2018). Nevertheless, there are few studies on ferroptosis and ferroptosis-related genes in PCa. We built the FPI in the current research to study the role of ferroptosis. As a result, we noted that the ferroptosis level was higher in tumors than in adjacent tissues in PCa, and FPI was related to BCR-free survival. This research divided all tumor samples into FPI-high group and FPI-low group. A conclusion was drawn that high FPI predicts poor BCR-free survival (Figures 1B,C).

The role of ferroptosis in PCa is still uncertain, but figuring out the relationship between mutation genes and FPI could promote our understanding of the functions of ferroptosis. TMB is regarded as a strong predictor of tumor behavior, characterized by microsatellite instability and defined as the mutation frequency of the tumor genome (Lv et al., 2020). To analyze the correlation between ferroptosis and gene mutations, the TMB value was calculated in the FPI-high group and FPI-low group, and the results showed that TMB was higher in the FPI-high group than in the FPI-low group (Figure 2C).

We performed GSEA analysis in both groups to research the association between the FPI and various cellular signaling pathways. It was identified that gene enrichment pathways were significantly different between the FPI-high and FPI-low groups (Figure 3). Some pathways in KEGG, such as cell adhesion molecules cams, complement, focal adhesion, coagulation cascades, and ECM receptor interaction, were usually enriched in the FPI-high group, whereas cardiac muscle contraction, glutathione metabolism, oxidative phosphorylation, and peroxisome pathways were enriched in the FPI-low group. As reported by previous studies, *GPX4* is an essential regulator of ferroptosis (Yang et al., 2014). Furthermore, ferroptosis is a mode of cell death characterized by iron-dependent lipid peroxidation (Dixon et al., 2012).

To further study the function of ferroptosis and FRGs in PCa, we screened FPI-associated DEGs in both the FPI-high and FPI-low groups. The function enrichment analysis of DEGs was carried out (Figure 4). GO analysis suggested that DEGs were involved in muscle system processes, muscle contraction, and striated muscle tissue development. Revealed by KEGG analysis, DEGs were related to neuroactive ligand–receptor interaction and hypertrophic cardiomyopathy. These functions are associated with ferroptosis. Univariate Cox regression analysis demonstrated that genes were significantly associated with BCR-free survival among these DEGs, and eight hub genes were screened out based on PPI network analysis (Figure 6). Then, Kaplan–Meier analysis was performed on these hub genes. Among them, the high-expressed gene (*SYT4*) and low-expressed genes (the other seven genes) had poor BCR-free survival in the FPI-high group. *SYT4* is a membrane protein, and ferroptosis is caused by the degradation of lipids that make up the cell membrane (Jia et al., 2020). It has been reported that *ACTC1* was upregulated in the skeletal muscle of PCa patients undergoing androgen deprivation therapy (ADT) as a compensatory response to ADT-induced muscle loss (Cheung et al., 2017).

Chemotherapy is one of the main ways to treat PCa, but the multidrug resistance of tumor cells often leads to chemotherapy failure. To further explore the relationship between drug sensitivity and ferroptosis, we discovered that the expression of hub genes may influence the sensitivity of numerous molecule drugs (Figure 7), and IC_50_ data of multiple drugs showed a negative correlation to the hub genes, implying that regulating cell ferroptosis could improve the therapeutic impact of tumor treatments. Ferroptosis has different effects on the drug sensitivity of different molecules; therefore, specific treatment should be given for specific PCa patients.

This study simply validated bioinformatics analysis results at the cellular level. LNCaP cells were treated with ferroptosis inducer erastin or ferroptosis inhibitor ferrostatin-1 to alter the ferroptosis level, and then the FPI was changed. Gene expression was detected by qRT-PCR and Western blot. Erastin upregulated the expression of SYT4 and downregulated the expression of the other four genes (ACTC1, ACTA1, ACTN2, and MYH6), and ferrostatin-1 led to the opposite results. Molecular experimental results were consistent with those of bioinformatics analysis, except for TNNI1, TNNC2, and NRAP.

This study revealed that ferroptosis is associated with gene mutations, various cellular signaling pathways, biochemical recurrence, and drug resistance in PCa. Thus, it should be beneficial to further research studies on the molecular mechanisms of ferroptosis. However, the current analysis is based on data at the RNA level, and the absence of data at the protein level renders the analysis potentially inaccurate. Therefore, this study also needs to be further updated and improved based on the continuous improvement of data.

## Data Availability

The datasets presented in this study can be found in online repositories. The names of the repository/repositories and accession number(s) can be found below: https://portal.gdc.cancer.gov/,TCGA.

## Supplementary Material

Supplementary Figure 1 Flowchart of TCGA-PRAD data analysis Supplementary Material. The R codes required for bioinformatics analysis.
