# Supplementary material for: Identification of Ferroptosis-Associated Genes in Prostate Cancer by Bioinformatics Analysis
Source: Front Genet. 2022 Jul 4;13:852565. doi: 10.3389/fgene.2022.852565 (PMC9289098; doi:10.3389/fgene.2022.852565)
Supplement: Supplementary file 1 [file DataSheet1.doc]

library(DESeq2)

library(GSVA)

library(ggplot2)

library(pheatmap)

library(clusterProfiler)

library(org.Hs.eg.db)

library(ggpubr)

library(ggbeeswarm)

library(ConsensusClusterPlus)

library(gelnet)

library(beeswarm)

library(EnhancedVolcano)

pheno <- read.csv('processedata/pheno.csv', header = TRUE, row.names = 1)

df.raw <- read.csv('processedata/counts.csv', header = TRUE, row.names = 1, check.names = FALSE)

result <- aggregate(x=df.raw[,1:551], by=list(df.raw$symbol), FUN=max, na.rm=T)

rownames(result) <- result$`Group.1`

df.counts <- result[-1, rownames(pheno)]

df.counts <- 2^df.counts - 1

write.csv(df.counts, 'processedata/df_counts.symbol.csv', quote=FALSE)

df.counts <- read.csv('processedata/df_counts.symbol.csv', header = TRUE, row.names = 1, check.names = FALSE)

keep <- rowSums(df.counts == 0) < 165

df.counts <- df.counts[keep,]

df.counts <- df.counts[rownames(pheno)]

pheno$sample_type.samples <- gsub("\\s", "_", pheno$sample_type.samples)

#DESeq2 analysis

dds <- DESeqDataSetFromMatrix(countData = round(df.counts),

colData = pheno,

design = ~ sample_type.samples)

table(pheno$sample_type.samples)

dds$sample_type.samples <- relevel(dds$sample_type.samples, ref = "Solid_Tissue_Normal")

dds <- DESeq(dds)

res <- results(dds)

res.sig <- subset(res, padj < 0.05)

dim(res.sig)

write.csv(res.sig, 'DEG_tumor_vs_norm.csv', quote=FALSE)

vsd <- vst(dds, blind=FALSE)

df.expr <- as.data.frame(assay(vsd))

expr <- df.expr[rownames(pheno)]

write.csv(df.expr, 'processedata/expr.csv', quote=FALSE)

expr <- read.csv('processedata/expr.csv', header = TRUE, row.names = 1, check.names = FALSE)

gset_hypoxia <- read.table('processedata/hypoxia_genes.txt', header = FALSE)

genelist_hypoxia <- list(hypoxia=gset_hypoxia$V1)

gset_angio <- read.table('processedata/geneset.txt', header = TRUE)

genelist_angio <- list(ANGIO=gset_angio$ANGIOGENESIS)

gene_positive <- list(Positive=c('LPCAT3','ACSL4','NCOA4','ALOX15','GPX4','SLC3A2','SLC7A11','NFE2L2','NOX1','NOX3','NOX4','NOX5'))

gene_negtive <- list(Negtive=c('FDFT1','HMGCR','COQ10A','COQ10B'))

gsva_postive <- gsva(as.matrix(expr), gene_positive, method='ssgsea', kcdf='Gaussian', abs.ranking=TRUE)

gsva_negtive <- gsva(as.matrix(expr), gene_negtive, method='ssgsea', kcdf='Gaussian', abs.ranking=TRUE)

FPI <- as.data.frame(t(gsva_postive - gsva_negtive))

gsva_hypo <- gsva(as.matrix(expr), genelist_hypoxia, method='ssgsea', kcdf='Gaussian', abs.ranking=TRUE)

gsva_angio <- gsva(as.matrix(expr), genelist_angio, method='ssgsea', kcdf='Gaussian', abs.ranking=TRUE)

df_hypo <- as.data.frame(t(gsva_hypo))

df_angio <- as.data.frame(t(gsva_angio))

pheno$hypo <- df_hypo$hypoxia

pheno$angio <- df_angio$ANGIO

pheno$FPI <- FPI$Positive

write.csv(pheno, 'processedata/pheno_gsva.csv')

pdata <- subset(pheno, sample_type.samples != 'Metastatic')

plot.data <- pdata[c('sample_type.samples', 'FPI')]

names(plot.data) <- c('Tissue', 'FPI')

ggplot(plot.data, aes(x=Tissue, y=FPI, col=Tissue)) +

geom_boxplot() +

geom_beeswarm() +

labs(x='') +

scale_color_manual(values=c('#ff4982', '#1fc7ff')) +

theme(axis.text.x = element_text(size=12),

axis.text.y = element_text(size=12),

axis.title.y = element_text(size=14),

legend.title = element_text(size=14),

legend.text = element_text(size=12)

) +

stat_compare_means(comparisons = list(c('Primary Tumor', 'Solid Tissue Normal')), label.y = c(0.3))

ggsave('result/PRAD_FPI.pdf')

ferrs <- c('LPCAT3','ACSL4','NCOA4','ALOX15','GPX4','SLC3A2','SLC7A11','NFE2L2','NOX1','NOX4','NOX5','FDFT1','HMGCR','COQ10A','COQ10B')

df_surv <- read.table('processedata/df_surv.txt', header = TRUE, row.names = 1, sep = '\t')

df_surv$gleason <- pheno[rownames(df_surv), 'gleason_score']

df_surv$PD-L1 <-

expr.ferrs <- expr[ferrs, rownames(df_surv)]

df_plot <- as.data.frame(t(expr.ferrs))

df_plot$FPI <- df_surv$FPI

df_plot$samples <- rownames(df_plot)

library(reshape2)

plot.info <- melt(df_plot, id=c('samples', 'FPI'), measure=names(df_plot)[1:15])

ggplot(plot.info, aes(x=variable, y=value, fill=FPI)) +

geom_violin() +

geom_boxplot(width=0.3, position=position_dodge(0.9)) +

xlab("") +

ylab("Relative expression") +

labs(fill='class') +

theme(axis.text.x = element_text(size=14, angle = 90),

axis.text.y = element_text(size=14),

axis.title.y = element_text(size=16),

legend.title = element_text(size=16),

legend.text = element_text(size=14)

) +

scale_fill_manual(values = c('#ff4982', '#1fc7ff')) +

stat_compare_means(aes(group = FPI),

label = "p.signif",

method="t.test", hide.ns=T)

ggsave('result/Ferr_genes.boxplot.pdf', width=12, height = 8)

expr_gsea <- expr[rownames(df_surv)]

expr_gsea$DESCRIPTION <- 1

gct <- expr_gsea[c('DESCRIPTION', rownames(df_surv))]

write.table(gct, 'processedata/expr.gct', quote=FALSE, sep='\t')

#DESeq2 for FPI

df_surv <- read.table('processedata/df_surv.txt', header = TRUE, row.names = 1, sep = '\t')

df_surv$sample_type <- pheno[rownames(df_surv), 'sample_type.samples']

df_tumor <- subset(df_surv, sample_type != 'Solid Tissue Normal')

counts_tumor <- df.counts[rownames(df_tumor)]

dds <- DESeqDataSetFromMatrix(countData = round(counts_tumor),

colData = df_tumor,

design = ~ FPI)

dds$FPI <- relevel(dds$FPI, ref = "LOW")

dds <- DESeq(dds)

res2 <- results(dds)

deg.sig <- subset(res2, abs(log2FoldChange) > 1 & padj < 0.05)

write.csv(deg.sig, 'result/deg.sig.csv', quote=FALSE)

pdf('result/DEG_volcano.pdf')

EnhancedVolcano(res2,

lab = '',

x = 'log2FoldChange',

y = 'padj',

pCutoff = 0.05, FCcutoff = 1, colAlpha = 0.6)

dev.off()

#GO and KEGG enrichment

gene.df <- bitr(mod_genes, fromType = "SYMBOL",

toType = "ENTREZID",

OrgDb = org.Hs.eg.db)

mod_genes <- c('SYT4','FRMPD4','MYH6','HMCN1','TNNI1','ACTN2','STMN2','PPFIA2','TMEM130','NRAP','VSTM2A','TNNC2','ACTC1','ACTA1','CCK','CKM','GNMT','DPYS','AKR1D1','PAH')

ego.cc <- enrichGO(gene = gene.df$ENTREZID,

OrgDb = org.Hs.eg.db,

ont = "CC",

pAdjustMethod = "BH",

pvalueCutoff = 0.05,

qvalueCutoff = 0.05)

dotplot(ego.cc)

ggsave('result/egoCC.pdf', width = 7, height = 10)

ego.bp <- enrichGO(gene = gene.df$ENTREZID,

OrgDb = org.Hs.eg.db,

ont = "BP",

pAdjustMethod = "BH",

pvalueCutoff = 0.05,

qvalueCutoff = 0.05)

dotplot(ego.bp)

ego.mf <- enrichGO(gene = gene.df$ENTREZID,

OrgDb = org.Hs.eg.db,

ont = "MF",

pAdjustMethod = "BH",

pvalueCutoff = 0.05,

qvalueCutoff = 0.05)

dotplot(ego.mf)

ggsave('result/egoMF.pdf', width = 7, height = 10)

kk <- enrichKEGG(gene.df$ENTREZID, organism="hsa", pvalueCutoff=0.05, pAdjustMethod="BH",

qvalueCutoff=0.1)

kks <- setReadable(kk, 'org.Hs.eg.db', 'ENTREZID')

ego <- enrichGO(gene = gene.df$ENTREZID,

OrgDb = org.Hs.eg.db,

ont = "ALL",

pAdjustMethod = "BH",

pvalueCutoff = 0.05,

qvalueCutoff = 0.05)

egox <- setReadable(ego, 'org.Hs.eg.db', 'ENTREZID')

write.csv(egox, 'result/modules_ego.csv', quote=FALSE)

write.csv(kks, 'result/modules_kegg.csv', quote=FALSE)

dotplot(kk)

#Cox regression

library(survival)

myfun <- function(x) {

cox.res <- coxph(Surv(df_tumor$days_to_BCR, df_tumor$BCR) ~ x)

summcph <- summary(cox.res)

p.value<-signif(summcph$wald["pvalue"], digits=2)

beta<-signif(summcph$coef[1], digits=2);#coeficient beta

HR <-signif(summcph$coef[2], digits=2);#exp(beta)

HR.confint.lower <- signif(summcph$conf.int[,"lower .95"], 2)

HR.confint.upper <- signif(summcph$conf.int[,"upper .95"],2)

HR <- paste0(HR, " (",

HR.confint.lower, "-", HR.confint.upper, ")")

res<-c(beta, HR, p.value)

names(res)<-c("beta", "HR (95% CI for HR)",

"p.value")

return(res)

}

deg_expr <- expr[rownames(deg.sig), rownames(df_tumor)]

cox_res <- apply(deg_expr, 1, myfun)

df_cox <- as.data.frame(t(cox_res))

cox.sig <- subset(df_cox, p.value < 0.05)

write.csv(cox.sig, 'result/cox.sig.csv', quote=FALSE)

cox.sig <- read.csv('result/cox.sig.csv', row.names = 1)

hubs <- c('STMN2','ACTC1','ACTA1','ACTN2','TNNI1','TNNC2','CCK','SYT4','FRMPD4','TMEM130','VSTM2A','PPFIA2','MYH6','NRAP','CKM','HMCN1')

df_surv <- read.table('processedata/df_surv.txt', header = TRUE, row.names = 1, sep = '\t')

hubs_expr <- as.data.frame(t(expr[hubs, rownames(df_surv)]))

df.tmp <- cbind(df_surv, hubs_expr)

write.csv(df.tmp, 'result/hubs_survdata.csv', quote=FALSE)

hub_fc <- as.data.frame(deg.sig)[hubs,]

write.csv(hub_fc, 'result/hubs_FC.csv', quote=FALSE)

gene.df <- bitr(hubs, fromType = "SYMBOL",

toType = "ENTREZID",

OrgDb = org.Hs.eg.db)

df_low <- subset(df_surv, FPI == 'LOW')

df_high <- subset(df_surv, FPI == 'HIGH')

expr_low <- expr[hubs, rownames(df_low)]

expr_high <- expr[hubs, rownames(df_high)]

write.csv(expr_low, 'processedata/hub_expr_low.csv', quote=FALSE)

write.csv(expr_high, 'processedata/hub_expr_high.csv', quote=FALSE)

cox.hubs <- cox.sig[hubs,]

write.csv(cox.hubs, 'processedata/hubs_cox.csv', quote=FALSE)

hubs <- c('ACTA1', 'ACTC1', 'ACTN2', 'MYH6', 'NRAP', 'SYT4', 'TNNC2', 'TNNI1')

pheno <- read.csv('processedata/pheno_gsva.csv', header = TRUE, row.names = 1)

hubs_epxr <- as.data.frame(t(expr[hubs, rownames(pheno)]))

dftmp <- cbind(pheno, hubs_epxr)

write.csv(dftmp, 'processedata/pheno_hubs.csv')
