# Supplementary figures and images for: Identification of Ferroptosis-Associated Genes in Prostate Cancer by Bioinformatics Analysis
Source: Front Genet. 2022 Jul 4;13:852565. doi: 10.3389/fgene.2022.852565 (PMC9289098; doi:10.3389/fgene.2022.852565)

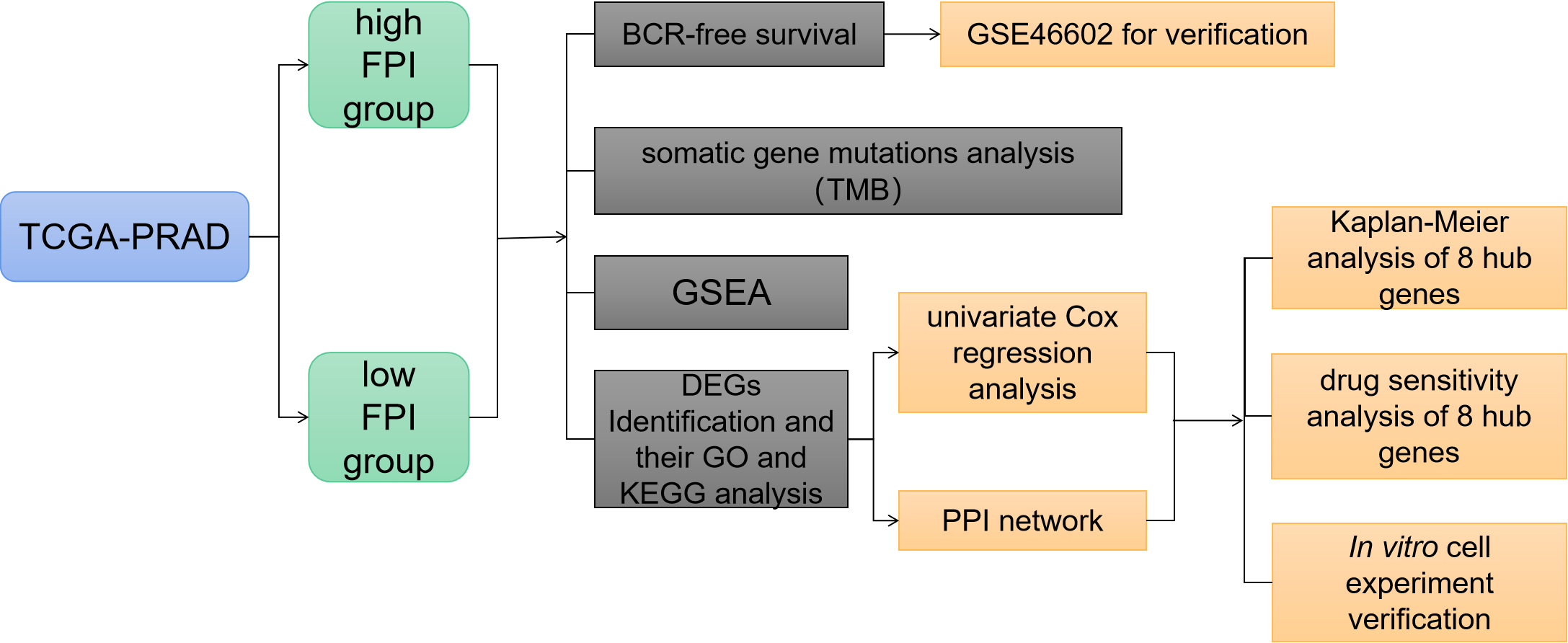

Supplement: Supplementary file 2 [file Image1.TIF]
